# A Critical Role for the Transient Receptor Potential Channel Type 6 in Human Platelet Activation

**DOI:** 10.1371/journal.pone.0125764

**Published:** 2015-04-30

**Authors:** Hari Priya Vemana, Zubair A. Karim, Christine Conlon, Fadi T. Khasawneh

**Affiliations:** Department of Pharmaceutical Sciences, College of Pharmacy, Western University of Health Sciences, Pomona, California, United States of America; University of Kentucky, UNITED STATES

## Abstract

While calcium signaling is known to play vital roles in platelet function, the mechanisms underlying its receptor-operated calcium entry component (ROCE) remain poorly understood. It has been proposed, but never proven in platelets, that the canonical transient receptor potential channel-6 (TRPC6) mediates ROCE. Nonetheless, we have previously shown that the mouse TRPC6 regulates hemostasis, thrombogenesis by regulating platelet aggregation. In the present studies, we used a pharmacological approach to characterize the role of TRPC6 in human platelet biology. Thus, interestingly, we observed that a TRPC6 inhibitor exerted significant inhibitory effects on human platelet aggregation in a thromboxane receptor (TPR)-selective manner; no additional inhibition was observed in the presence of the calcium chelator BAPTA. This inhibitor also significantly inhibited human platelet secretion (dense and alpha granules), integrin IIb-IIIa, Akt and ERK phosphorylation, again, in a TPR-selective manner; no effects were observed in response to ADP receptor stimulation. Furthermore, there was a causal relationship between these inhibitory effects, and the capacity of the TRPC6 inhibitor to abrogate elevation in intracellular calcium, that was again found to be TPR-specific. This effect was not found to be due to antagonism of TPR, as the TRPC6 inhibitor did not displace the radiolabeled antagonist [^3^H]SQ29,548 from its binding sites. Finally, our studies also revealed that TRPC6 regulates human clot retraction, as well as physiological hemostasis and thrombus formation, in mice. Taken together, our findings demonstrate, for the first time, that TRPC6 directly regulates TPR-dependent ROCE and platelet function. Moreover, these data highlight TRPC6 as a novel promising therapeutic strategy for managing thrombotic disorders.

## Introduction

Platelets are anucleate cells that play an important role in hemostasis and thrombosis [1]. With regards to platelet activating agents, thromboxane A_2_ (TXA_2_) is one of the most studied platelet agonists. Studies on platelets demonstrated that TXA_2_ signals [2, 3], at least in part, through the regulation of cellular calcium upon binding to its G Protein Coupled Receptor (GPCR), i.e., known as the thromboxane receptor (TPR) [4–7]. It is now clear that TPR controls additional aspects of cellular function, specifically through coupling to multiple G-proteins (GPs) including G_q_ [8–10], G_13_ [11–13], and G_i_ [14–17]. Nonetheless, functional and physical coupling of platelet TPRs has only been documented with two GPs, i.e., G_q_ [8, 16] and G_13_ [11, 16], with the G_q_-PLCβ-inositol triphosphate (IP_3_)-Ca^2+^-signaling cascade being the most characterized of the two. In this regard, experiments by Offermanns group have provided evidence that platelet shape change can be stimulated through G_12/13_ pathway [18]. Specifically, it was shown that TPR-mediated platelet shape change was still observed in mice deficient in G_q_ [19], whereas the aggregation response was lacking [20]. This suggestion would seem to be consistent with earlier observations that TPR agonists can induce platelet shape change in the absence of measurable intracellular calcium levels (which is presumably a G_q_-mediated event) [7, 21]. Changes in intracellular calcium [22–24] have been shown to play essential roles in the initial activation of platelets and the recruitment of feedback signaling mechanisms such as ADP secretion [25, 26]. In turn, these feedback mechanisms (ADP) will initiate separate GPCR-signaling in platelets that also involve calcium entry. While research efforts have attempted to define the channels involved in the G_q_-dependent, receptor-operated calcium entry (ROCE) and store-operated calcium entry (SOCE) [27], the underlying mechanism at the molecular level, especially that for ROCE, is still poorly understood. In this regard, the transient receptor potential channel (TRPC) proteins, were suggested to be mostly receptor-activated, and hence an ideal candidate for ROCE [28].

Studies on the expression profile of TRPCs in platelets indicate that platelets express low levels of TRPC1 [29] that is mostly found in the intracellular membrane, and high levels of TRPC6 that is exclusively found in the plasma membrane [30, 31]. Regarding the role of TRPC1 in platelet function, experiments using the TRPC1 knockout (KO) platelets revealed that these platelets displayed fully intact SOCE, unaltered calcium homeostasis, and intact *in vitro and in vivo* platelet function [29]. Based on these considerations, clearly the role of TRPC6 in platelet function warrants investigation. To this end, our own findings [32] using TRPC6 KO mice have shown that TRPC6 plays a critical role in physiological hemostasis and thrombogenesis. These defects were attributed to defective platelet aggregation, downstream of TPR. However, whether TRPC6 regulates ROCE, and plays a critical role in human platelet function remains unknown. To a large extent, this derived, from lack of pharmacological tools or inhibitors to study the role of TRPC6 channels in human platelets. Thus, our current work utilized a new TRPC6 inhibitor to characterize its role in platelet function. Our findings demonstrated, for the first time, that TRPC6 mediates ROCE, thereby regulating platelet aggregation, secretion, integrin exposure, as well as Akt and ERK phosphorylation, and that, interestingly, it does so in a TPR-dependent/selective manner. Importantly, these effects did not derive from TPR antagonism by the TRPC6 inhibitor, as the latter could not displace the antagonist [^3^H]SQ29,548 from its TPR binding sites. Of note, when the TRPC6 inhibitor was combined with BAPTA, the calcium chelator, no additional inhibition of aggregation was observed beyond that observed with BAPTA alone, i.e., its inhibitory effects could no longer be observed. Finally, we also found that TRPC6 is involved in clot retraction and contributes to hemostasis and thrombosis.

## Materials and Methods

Human blood studies were approved by the Institutional Review Board (IRB) at Western University of Health Sciences, Pomona, CA, and donors were asked to sign a written consent, and a subjects’ bill of rights, that were previously approved by the IRB.

### Reagents

U46619 was from Cayman Chemical (Ann Arbor, MI). Thrombin receptor activating peptide 4 (TRAP4; AYPGKF-NH2) and ADP analog Adenosine 5′-[β-thio]diphosphate trilithium salt was from Sigma Aldrich (St. Louis, MO). Antibodies for Akt, pAkt, ERK and pERK were from Cell Signaling (Danvers, MA). ADP and other platelet disposables were from Chrono-Log (Havertown, PA). TRPC6 inhibitor (GsMTx-4) was from Alomone labs (Israel). FITC-conjugated Annexin V, anti–P-selectin, and PAC-1 antibodies were purchased from Cell Signaling Technology, Inc. (Danvers, MA). Fura-2 acetoxymethyl ester (fura-2/AM) and Pluronic F-127 were from Invitrogen (Grand Island, NY). Sodium citrate, whatman filter paper, ferric chloride, sodium chloride, potassium chloride, sodium dihydrogen phosphate, magnesium chloride, sodium bicarbonate, and D-dextrose were from Fisher Scientific (Hanover Park, IL). The radiolabeled [^3^H]SQ29,548 was purchased from PerkinElmer (Waltham, MA). BAPTA was purchased from Tocris Bioscience (Ellisville, Missouri).The C57BL/6 mice were obtained from Jackson laboratory (Bar Harbor, ME). Platelet count was determined using an automated hematology analyzer (Drew Scientific Dallas, TX).

### Animals

C57BL/6 J mice were from Jackson Laboratories (Bar Harbor, ME). All mice used for experiments were 8–10 weeks of age. All experiments involving animals were performed in compliance with the relevant laws and institutional guidelines, and were approved by the Western University of Health Sciences Institutional Animal Care and Use Committee.

### Preparation of Human Platelets

Blood was drawn from healthy volunteers who denied taking any medication for 1 week prior to collection. Coagulation was inhibited by 3.8% w/v sodium citrate solution (1 part sodium citrate to 9 parts blood). Human platelet rich plasma (PRP) was obtained by centrifugation at room temperature. Platelets were counted with automated hematology analyzer and their count was adjusted to 7 x 10^7^ platelets/ml, prior to each experiment. Washed human platelets were prepared as we described [33]. PRP was isolated in the presence of 0.37 U/ml apyrase and 10 ng/ml PGI_2_ by centrifugation at 150 × g for 10 min at 20°C. PRP was centrifuged at 900 × g for 10 min and pelleted platelets were resuspended in HEPES/Tyrode's buffer (20 mM HEPES/NaOH, pH 6.5, 128 mM NaCl, 2.8 mM KCl, 1 mM MgCl_2_, 5 mM D-glucose, 12 mM NaHCO_3_, 0.4 mM NaH_2_PO_4_) containing 1 mM EGTA, 0.37 U/ml apyrase, and 10 ng/ml PGI_2_. Platelets were washed and resuspended in HEPES/Tyrode's buffer (pH 7.4) without EGTA, apyrase, or PGI_2_. The final platelet count was adjusted to 4 × 10^8^ platelets/ml, unless otherwise indicated. PRP was isolated in the presence of apyrase (0.37 U/ml) and PGI_2_ (10 ng/ml) by centrifugation at 150 × g for 10 min at RT. PRP was centrifuged at 900 × g for 10 min and platelets were resuspended in HEPES/Tyrode’s buffer containing 1 mM EGTA, apyrase, and PGI_2_. Platelets were washed and resuspended in HEPES/Tyrode's buffer (pH 7.4) without EGTA, apyrase, or PGI_2_.

### In vitro Platelet Aggregation and Secretion

PRP was incubated with TRPC6 inhibitor (10 μM) for 5 min prior to experiments, except in control experiments. Platelets were activated with ADP (5 μM) and U46619 (0.5 μM) in the presence or absence of TRPC6 inhibitor (10 μM). Platelets were also activated with U46619 (0.5 μM) in the presence and absence of the TRPC6 inhibitor (10 μM) with or without the addition of BAPTA (10 μM). Platelet aggregation was measured by the turbidometric method using model 490 aggregometer (Chrono-Log Corporation, Havertown, PA). ATP secretion was monitored in parallel with platelet aggregation after addition of the luciferin-luciferase (1% [v/v] final concentration) reagent to the same PRP suspension used for aggregation and measured for ATP secretion upon adding 1 μM U46619 and 0.1 U/ml thrombin. Each experiment was repeated at least 3 times, with blood collected from three different human donors.

### Flow Cytometric Analysis

Flow cytometric analysis was carried out as we described [34]. Human platelets (2 × 10^8^) were incubated in the presence or absence of TRPC6 inhibitor (10 μM) for 5 minutes and then stimulated with thrombin (0.1 U/ml), U46619 (0.5 μM) for 3 minutes. The reactions were stopped by fixing the platelets with 2% formaldehyde for 30 min at room temperature. Platelets were incubated with FITC-conjugated anti–P-selectin or PAC-1 antibodies at room temperature for 30 min in the dark. Finally, the platelets were diluted 2.5 fold with HEPES/Tyrode buffer (pH 7.4). The samples were transferred to FACS-tubes and fluorescent intensities were measured using a BD Accuri C6 flow cytometer and analyzed using CFlow Plus (BD Biosciences, Franklin Lakes, NJ).

### Measurement of Intracellular Calcium in Platelets

Intra-platelet calcium was measured using Fura-2-acetoxymethyl ester (Fura-2AM) as described [33]. Human platelets (2.0 x 10^8^/ml) were labeled with 12.5 μM Fura-2AM and 0.2% Pluronic F-127 in HEPES/Tyrode buffer (pH 7.4) for 45 min at 37°C. After washing, the platelets were resuspended without apyrase to a concentration of 2.0 x 10^8^/ml. Samples (1 ml) were added to siliconized cuvettes, recalcified with 0.7 mM CaCl_2_, and incubated in the presence or absence of TRPC6 inhibitor (10 μM) for 5 min and then stimulated with OAG (150 μM) for 3 min with constant stirring. Fluorescence was analyzed by excitation at 340 nm and 380 nm and emission was measured at 509 nm using a model LS50B Luminescence Spectrometer (Perkin-Elmer Instruments, Shelton, CT). The ratio of fura-2 emissions were calculated simultaneously using FL WinLab software and converted to [Ca^2+^]_i_, as described previously [35].

### Immunoblotting

Immunoblotting was carried out as described [33]. Briefly, human platelets were incubated in the presence or absence of TRPC6 inhibitor (10 μM) for 5 minutes and then stimulated with U46619 (0.5 μM) and ADPβS (100 μM) for 3 minutes followed by lysis with 1 × lysis buffer. Proteins were separated by sodium dodecyl sulfate-polyacrylamide gel electrophoresis (SDS-PAGE) and transferred to Immobilon-P PVDF membranes (Bio-Rad, Hercules, CA). They were then probed with the primary antibodies (ERK, pERK, Akt and pAkt) and visualized with horseradish peroxidase-labeled anti-rabbit or anti-mouse IgG as required. The antibody binding was detected using enhanced chemiluminescence substrate (Thermo Scientific, Rockford, IL). Images were obtained with ChemiDoc MP Imaging System (Bio-Rad, Hercules, CA) and quantified with Image Lab software Version 4.1 (Bio-Rad, Hercules, CA).

### Fibrin Clot Retraction Assay

With slight modification fibrin clot retraction assay was performed as discussed in Osdoit and Rosa [36]. Briefly, whole blood was collected and washed platelets were isolated as discussed above. CaCl_2_ was added extemporaneously, at a final concentration of 1 mM. Glass tubes designed for aggregation were used for retraction assays. First, a 10% (w/v) polyacrylamide cushion was polymerized at the bottom of the tubes to avoid clot adherence. Tubes were then rinsed extensively in distilled water. Washed platelets resuspended at 1×10^8^/ml in Hepes-Tyrode buffer (pH 7.4). Platelets were incubated with TRPC6 inhibitor (10 μM) for 5 min. Fibrinogen (500 μg/ml) was added in 0.5 ml platelets aliquots, and clot retraction was initiated by quickly adding thrombin (0.1 U/ml). The reaction was transferred to the glass tube and the reaction was set at room temperature. Pictures were taken at time intervals of 10 min up to one hour using a digital camera.

### Binding Assay

These experiments were conducted as we described before [37]. Resuspended platelets were prepared as we described previously [38]. The platelet suspension (1 x10^9^ platelets/ml) was incubated with the radiolabeled [^3^H]SQ29,548 (1 nM) at RT for 10 min, and then increasing concentrations of the displacing TRPC6 inhibitor (10–50 μM) were added for an additional 45 min. The [^3^H]SQ29,548 bound platelets were captured by running through 0.45 mm Millipore filter over a vacuum suction unit. The filters were then washed once and counted for radioactivity in a Beckman LS 6000 liquid scintillation counter.

### Tail bleeding time assay

Mice 8–10 weeks old were injected with and without TRPC6 inhibitor and the bleeding time was recorded one-hour post injection. Hemostasis was measured using the tail transection technique [34]. Briefly, mice were anesthetized using isoflurane and then placed on a 37°C heating blanket (Harvard Apparatus Limited, Edenbridge, KY, USA) before the tail was transected with a clean cut using a sterile scalpel at a distance of 5 mm from the tip. After transection, the tail was immediately immersed in saline maintained at 37°C, constant temperature. Bleeding was observed visually and recorded as the time from the tail transection to the moment the blood flow stopped and did not resume within 60 s from the initial cessation time. Normal bleeding times for murine specimens lasts between 1–3 min. However, when bleeding did not stop within 15 min, pressure was applied to the tail and styptic powder was used to help close the wound, thus avoiding excessive loss of blood. Bleeding times beyond 15 min were considered as the cut-off time for the purpose of statistical analysis.

### *In vivo* thrombosis model

These studies were performed as described previously [34, 39]. Mice that are 8–10 weeks old were injected with and without TRPC6 inhibitor. Animals were anesthetized and placed on a 37°C heated surgical table under a stereo microscope (Leica Microsystems Ltd, CH-9435, Heerbrugg, Switzerland). A midline incision of the skin was made directly on top of the right common carotid artery region, and a segment of the left common carotid artery was exposed and cleaned. Baseline carotid blood flow was measured and recorded with a miniature Doppler flow probe (Model 0.5 VB, Transonic System, Ithaca, NY, US), interfaced with a flowmeter (model TS402, Transonic Systems). Thrombosis was induced by applying a saturated segment of filter paper (0.5 × 1 mm) in 7.5% ferric chloride, onto the carotid artery. After 3 min of exposure, the filter paper was removed. The carotid blood flow was continuously monitored for 45 min after ferric chloride application, and the data was registered by a computerized data acquisition program (LabChart6, ADInstruments, Colorado Springs, CO, and USA). Time to occlusion was calculated as the difference in time between the removal of the filter paper and stable occlusion, which was described as zero blood flow for 2 min. An occlusion time beyond 45 min was considered as the cut-off time for the purpose of statistical analysis.

### Statistical Analysis

All experiments were performed at least three times. Analysis of the data was performed using GraphPad PRISM statistical software (San Diego, CA) and presented as mean ± SEM. The Mann-Whitney test was used for the evaluation of differences in mean occlusion and bleeding times. Analysis was also conducted using t-test, and similar results were obtained. Significance was accepted at P<0.05 (two-tailed P value), unless stated otherwise.

## Results

### Effect of TRPC6 inhibitor on platelet Aggregation

**Fig 1:**
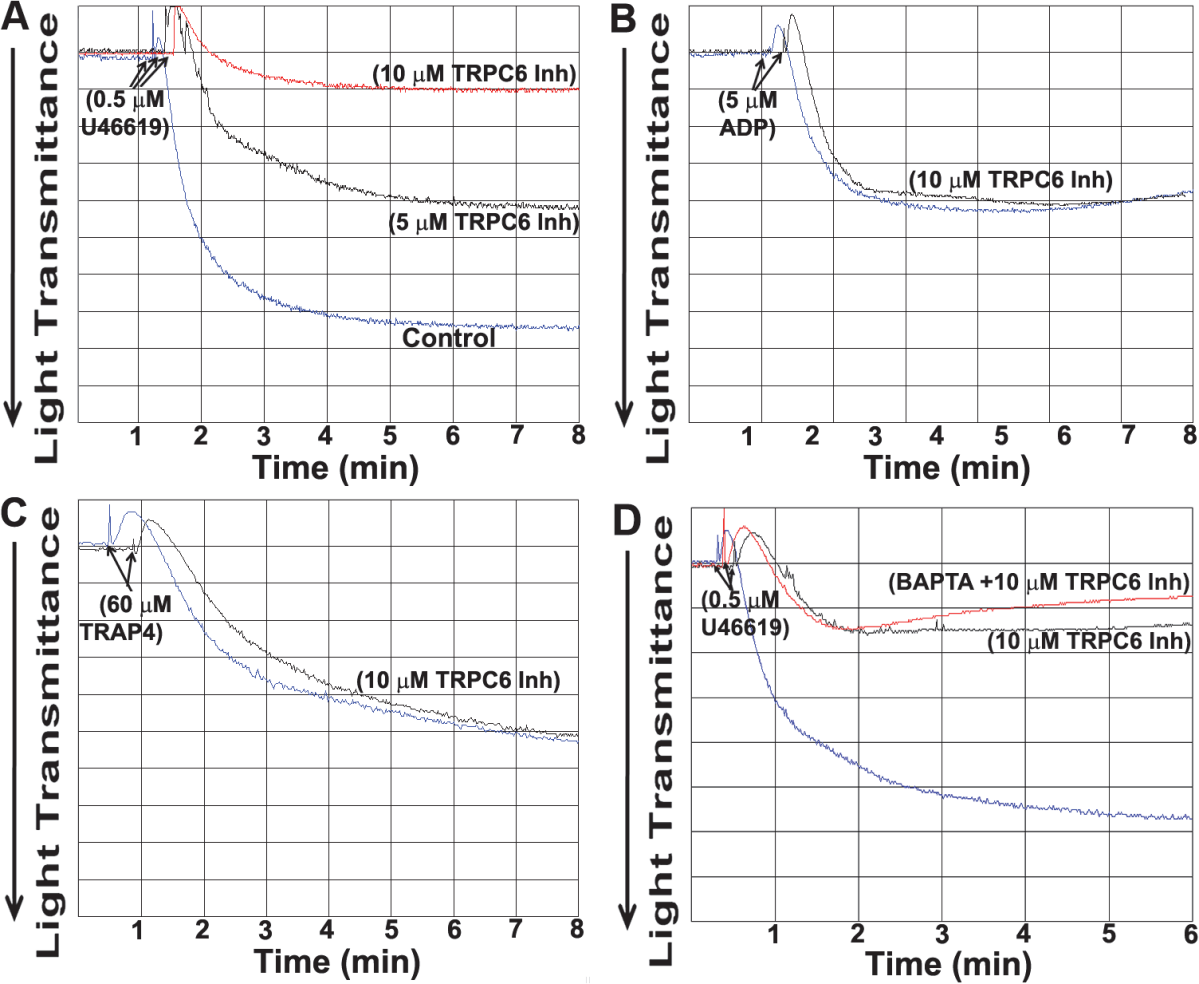
Effect of TRPC6 inhibitor on human platelet aggregation. PRP was incubated without or with the TRPC6 inhibitor (5 μM and/or 10 μM) for 5 min followed by stimulation with (A) U46619 (0.5 μM), (B) ADP (5 μM), (C) TRAP4 (60 μM), or (D) U46619 (0.5 μM) with or without BAPTA (10 μM). Each experiment was repeated three times, with three separate donors.

In order to evaluate the role of the TRPC6 in platelet function downstream of TPRs and other G_q_-coupled receptors, TRPC6 inhibitor (pharmacological approach) was employed in our studies. Our initial experiments revealed that the 5 μM of the TRPC6 inhibitor exerted significant inhibitory effect on platelet aggregation induced by 0.5 μM of the TPR agonist U46619, when compared to vehicle control (Fig 1). This inhibitory effect was found to be dose (10 μM) dependent (Fig 1). This is the first demonstration that the mechanism by which TPRs stimulate platelet aggregation involves TRPC6. On the other hand, there was no difference in the aggregation response between TRPC6 inhibitor-, and control-treated platelets when they are stimulated by either 5 μM ADP or 60 μM of TRAP4 (Fig 1). These results indicate that the inhibition of aggregation in the presence of the TRPC6 inhibitor is agonist/receptor (i.e., TPR)-specific. In addition, to assess the specificity of TRPC6 inhibitor we first treated with BAPTA to chelate extracellular calcium. As one might expect, when the TRPC6 inhibitor was combined with BAPTA, no additional inhibition of aggregation was detected beyond that observed with BAPTA alone (Fig 1), i.e., its inhibitory effects could no longer be observed. This finding is consistent with the notion that TRPC6 mediates calcium entry and further argues for the specificity of this inhibitor.

### Effect of TRPC6 inhibitor on glycoprotein IIb-IIIa activation

**Fig 2:**
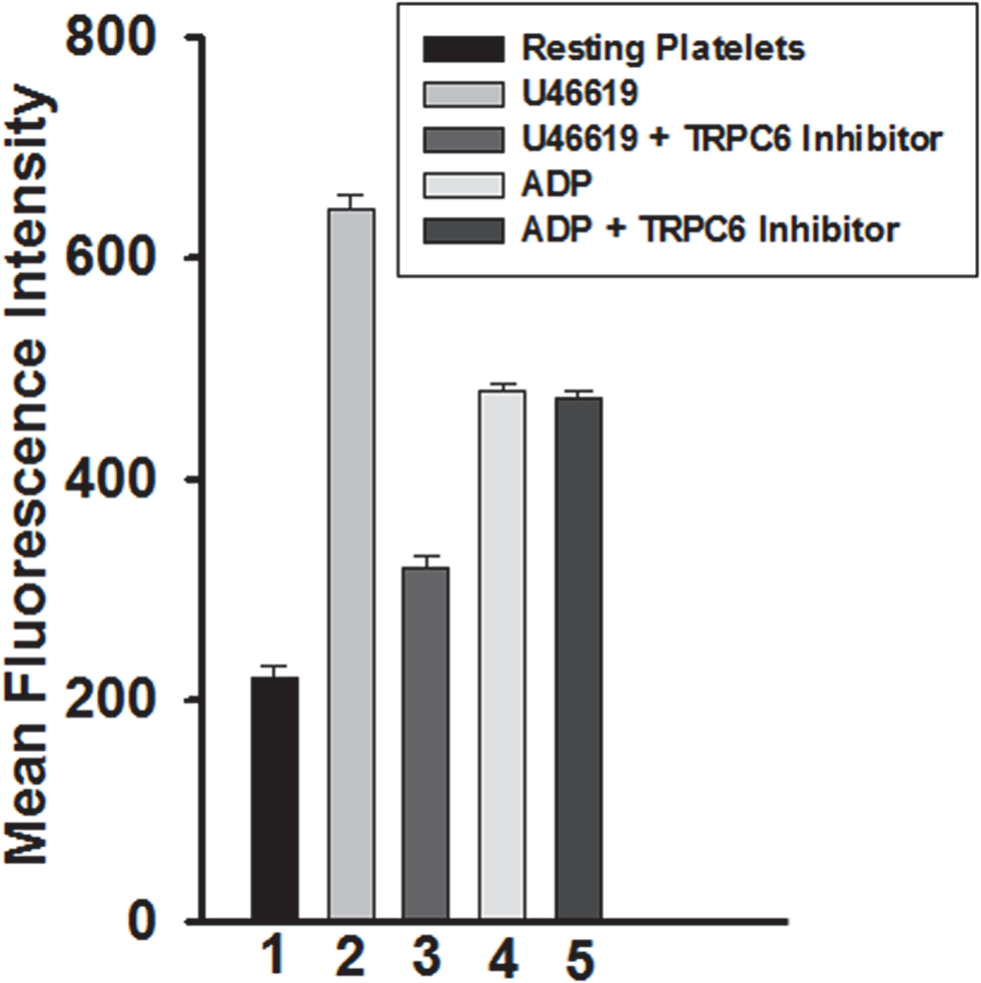
Effect of TRPC6 inhibitor on glycoprotein IIb-IIIa activation *in vitro*. Washed platelets were incubated in the presence or absence of TRPC6 inhibitor (10 μM) for 5 minutes and then stimulated with U46619 (0.5 μM) and ADP (5 μM) for 3 minutes. The reactions were stopped by fixing the platelets with 2% formaldehyde for 30 min at room temperature. Platelets were incubated with FITC-conjugated PAC-1 antibody, the fluorescent intensities were measured by flow cytometry, and the data were plotted as histogram. Each experiment was repeated at least four times, with blood obtained from four separate donors (n = 4; P < 0.01, Mann-Whitney test).

Next, we sought to investigate whether the inhibition of aggregation, would be accompanied by inhibition of GPIIb-IIIa activation. Indeed, we observed significant blockade of U46619 (0.5 μM)-induced activation of GPIIb-IIIa, in (10 μM) TRPC6 inhibitor-treated platelets, compared to control (Fig 2). Conversely, we did not detect any inhibition in GPIIb-IIIa in response to 5 μM of the agonist ADP (Fig 2).

### Effect of TRPC6 inhibitor on platelet dense and alpha granule secretion

**Fig 3:**
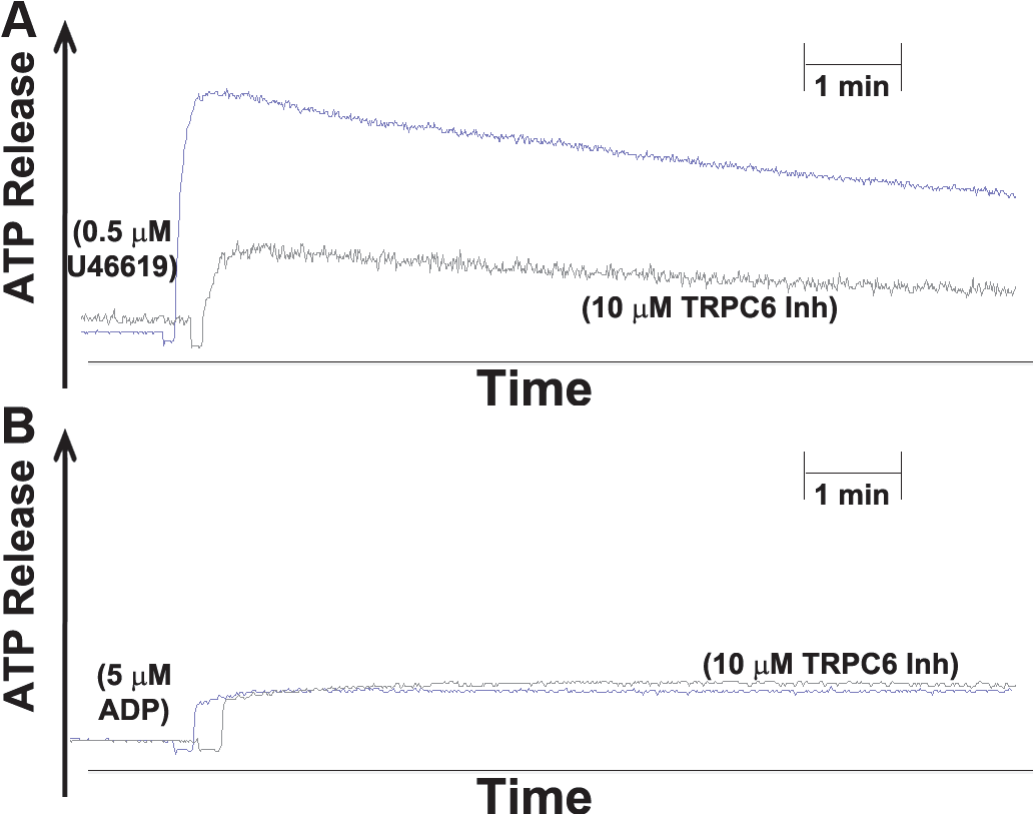
Effect of TRPC6 inhibitor on human platelet ATP secretion stimulated by U46619. PRP was incubated without or with TRPC6 inhibitor (10 μM) for 5 min followed by stimulation of PRP with (A) U46619 (0.5 μM), or (B) ADP (5 μM). Each experiment was repeated three times, with three separate donors.

**Fig 4:**
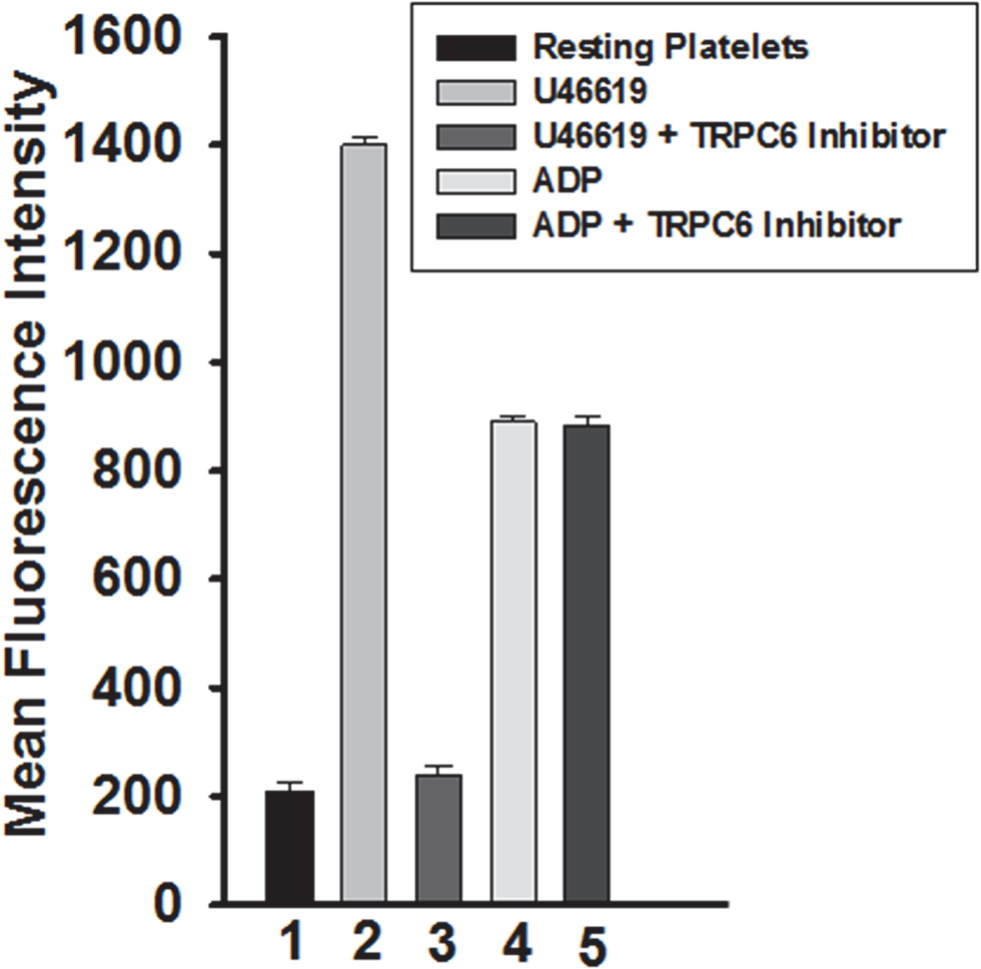
Effect of TRPC6 inhibitor on alpha granule secretion (P-selectin expression). Washed platelets were incubated in the presence or absence of TRPC6 inhibitor (10 μM) for 5 minutes and then stimulated with U46619 (0.5 μM) and ADP (5 μM) for 3 minutes. The reactions were stopped by fixing the platelets with 2% formaldehyde for 30 min at room temperature. Platelets were incubated with FITC-conjugated anti–P-selectin antibody, the fluorescent intensities were measured by flow cytometry, and the data were plotted as histogram. Each experiment was repeated at least four times, with blood obtained from four separate donors (P < 0.01, Mann-Whitney test).

We next assessed the effect of the TRPC6 inhibitor on platelet secretion (dense and alpha granules). It was found that 0.5 μM U46619-induced ATP release, a measure of dense granule secretion, was inhibited when the platelets were incubated with 10 μM of the TRPC6 inhibitor (Fig 3). The U46619-triggered alpha granule secretion analyzed by flow cytometry of P-selectin also was inhibited in platelets treated with TRPC6 inhibitor (Fig 4). In contrast, and as one might expect in light of the aggregation and GPIIb-IIIa data, the TRPC6 inhibitor did not exert any apparent inhibitory effects on 5 μM ADP-induced dense and alpha granule secretion (Fig 3 and Fig 4). This data combined with the GPIIb-IIIa activation indicate that TRPC6 inhibitor has the capacity to inhibit expression of multiple markers of platelet activation.

### Effect of TRPC6 inhibitor on intracellular calcium

**Fig 5:**
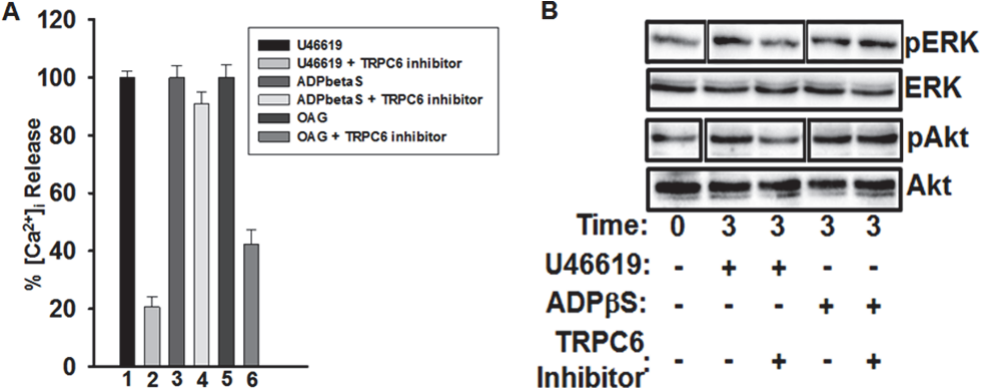
Effect of TRPC6 inhibitor on intracellular calcium in human platelets *in vitro* and on ERK and Akt phosphorylation. (A) Human platelets were loaded with Fura-2/AM to measure intracellular [Ca^2+^]_i_, in the presence or absence of TRPC6 inhibitor (10 μM), and activated with U46619 (0.5 μM), ADPβS (100 μM) and OAG (150 μM). Each experiment was repeated at least three times, with blood obtained from three separate donors. (B) Human platelets were incubated in the presence or absence of TRPC6 inhibitor (10 μM) for 5 minutes and then stimulated with U46619 (0.5 μM), ADPβS (100 μM) for 3 minutes, and subjected to immunoblotting with ERK, pERK, Akt and pAkt antibodies.

In this set of experiments we investigated the role of TRPC6 in ROCE, in order to confirm that the aforementioned “defects” in platelet function are causally related to inhibition of calcium homeostasis/entry by the TRPC6 inhibitor. As expected, the TRPC6 inhibitor (10 μM) significantly lowered intracellular calcium level in response to the TPR agonist U46619 (0.5 μM; Fig 5A), but was without any apparent effects when platelets were stimulated by 100 μM of the stable ADP analog Adenosine 5′-[β-thio] diphosphate trilithium salt (ADPβS; Fig 5A). To further confirm these findings, we next utilized a membrane permeable analogue of the G_q_ activator diacylglycerol (DAG), namely 1-oleoyl-2-acetyl-sn-glycerol (OAG), which is previously shown to be an agonist for TRPC6. Our measurements revealed that TRPC6 inhibitor (10 μM) produced significant inhibition on 150 μM OAG-induced ROCE, when compared to the control (Fig 5A). To biochemically confirm our findings; we next examined the effect of the TRPC6 inhibitor on ERK and Akt phosphorylation, which are known to be downstream of TPRs. It was found that incubation with 10 μM of the TRPC6 inhibitor, ameliorated phosphorylation of ERK and Akt upon stimulation with 0.5 μM U46619, but not with ADPβS (Fig 5B).

### Effect of the TRPC6 inhibitor on binding of the radiolabelled TPR antagonist [^3^H]SQ29,548

**Fig 6:**
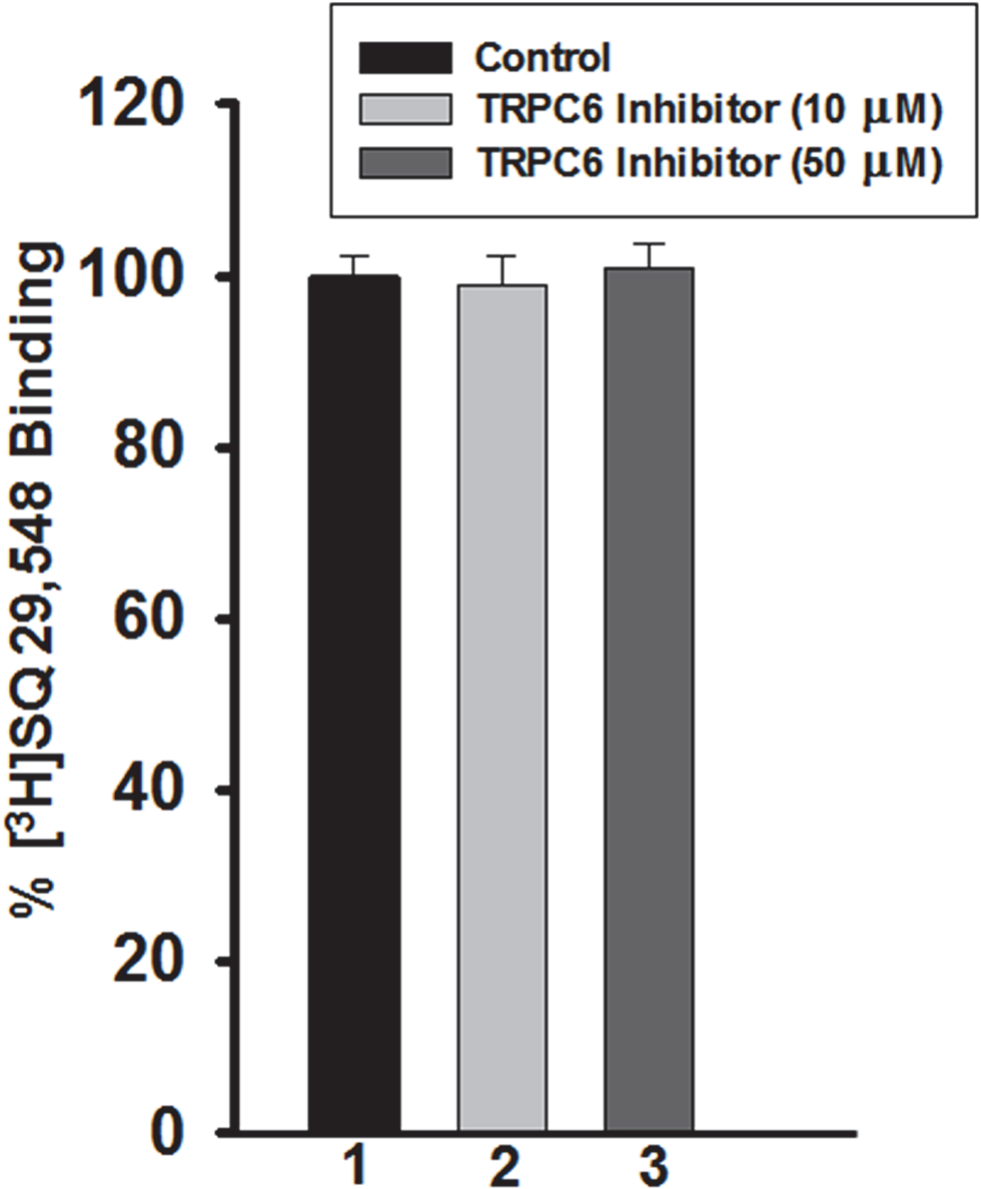
Effect of the TRPC6 inhibitor on binding of the radiolabelled TPR antagonist [^3^H]SQ29,548. Binding displacement of 1 nM [^3^H]SQ29,548 with increasing concentrations of the TRPC6 inhibitor (10–50 μM), in human platelets (n = 3; P < 0.01, Mann-Whitney test).

In order to further define the molecular mechanism by which the TRPC6 inhibitor exerts its inhibitory effects on platelets, a displacement radiolabeled ligand binding assay was performed. Thus, platelets were incubated with the radiolabeled TPR antagonist [^3^H]SQ29,548, and increasing concentrations of TRPC6 inhibitor (10–50 μM) were added. The TRPC6 inhibitor, at a concentration that almost completely inhibited aggregation (10 μM; Fig 6) did not appear to displace [^3^H]SQ29,548 from its TPR binding sites. In fact, no ligand displacement was observed even with a TRPC6 inhibitor concentration as high as 50 μM (Fig 6). These findings indicate that the mechanism by which TRPC6 inhibitor exerts its effects on platelets does not involve TPR antagonism.

### Effect of TRPC6 inhibitor on clot retraction

**Fig 7:**
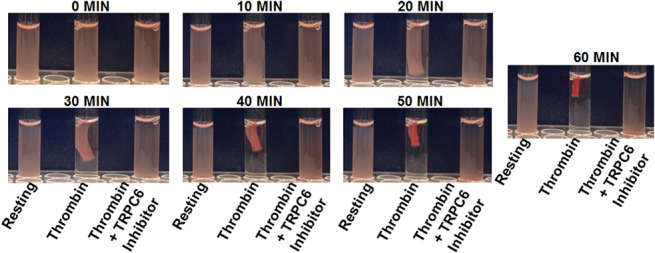
Effect of TRPC6 inhibitor on clot retraction. Clot retraction of washed platelets was intiated by adding thrombin (0.1 U/ml) in the presence or absence of TRPC6 inhibitor (10 μM) and photographed for every 10 minutes up to one hour (n = 4).

To test the possibility that TRPC6 may regulate the ability of platelets to generate contractile forces, we analyzed the effect of the TRPC6 inhibitor on clot retraction. Indeed, it was found that the TRPC6 inhibitor (10 μM) significantly inhibited clot retraction, when compared with the control (Fig 7), indicating that TRPC6 affects does play a role in integrin outside-in signaling [40, 41].

### Effect of TRPC6 inhibitor on hemostasis

**Fig 8:**
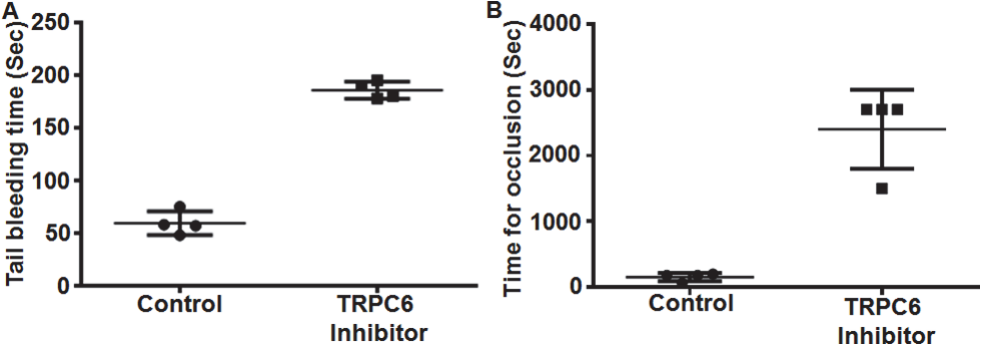
Effect of TRPC6 inhibitor tail vein injections on mouse hemostasis following tail tip excision and on the time for occlusion in a carotid artery injury thrombosis model. Mice were injected with TRPC6 inhibitor (10 μM) or vehicle 1 hour before their tail bleeding times and time for occlusion are measured. TRPC6 inhibitor treatment resulted in a significant increase in bleeding time (n = 3; P < 0.01, Mann-Whitney test) (A) and significantly prolonged time for occlusion (n = 3; P < 0.01, Mann-Whitney test) (B). Each point represents a single animal.

Given the established role of Ca^2+^ signaling in platelet biology, and our present findings that TRPC6 regulates (TPR-mediated) *in vitro* platelet function, we next investigated whether ROCE/TRPC6 is involved in hemostasis (i.e., in *in vivo* platelet function). Our studies revealed that mice injected with 10 μM of the TRPC6 inhibitor exhibited a significantly prolonged tail bleeding time, in comparison to the mice injected with vehicle control (Fig 8A).

### Effect of TRPC6 inhibitor on thrombus formation

In the last set of studies, we examined the contribution of TRPC6 to thrombogenesis, using a FeCl_3_-induced carotid artery thrombosis model. As might be expected given the role of TPRs in thrombus formation, mice treated with the TRPC6 inhibitor (10 μM) exhibited a significantly prolonged time for occlusion, when compared with the control (Fig 8B). Therefore, this finding indicates that the TRPC6 inhibitor protects against the development of thrombotic events *in vivo*, supporting the notion that TRPC6 contributes to thrombotic disease states.

## Discussion

Although calcium signaling has been shown to play an important role in platelet function, the molecular mechanism underlying its receptor-operated component (i.e., ROCE) is still poorly understood. It has been proposed, though never proven, that transient receptor potential channels participate in ROCE and are therefore directly involved in platelet activation. Since calcium signaling plays an important role in *in vivo* platelet activation and thrombus formation and given that most if not all agonists signal through regulation of cellular calcium, investigating the role of TRPC6 in TPR, and other G_q_-coupled receptors, such as ADP (i.e., P2Y_1_) and PAR4, seems clearly warranted. We have previously shown using a genetic deletion approach in mice, that the mouse TRPC6 regulates platelet aggregation, and is involved in hemostasis and thrombogenesis [32]. However, whether TRPC6 plays a role in human platelet function, species-specific differences, remains to be investigated. To this end, in human platelets, TRPC6 has been reported to be highly expressed as compared to other TRPC isoforms. Based on these considerations, we propose that TRPC6 plays an important role in intracellular-calcium changes (ROCE) and platelet activation, downstream of TPRs and other G_q_-coupled receptors. In order to address this issue, we employed a pharmacological inhibitor of TRPC6. Thus, we found that the TRPC6 inhibitor treated platelets exhibited an impaired aggregation, GPIIb-IIIa activation, dense and alpha granule secretion, in response to TPR agonist stimulation. However, there was no apparent difference between TRPC6 inhibitor, and control-treated platelets, when stimulated by ADP (or TRAP4). These findings support the notion, that TPR-mediated human platelet function is dependent on TRPC6, and that the latter’s role in platelets is receptor/agonist specific. Of note, the defect in TPR-mediated aggregation is consistent with that we observed in TRPC6 KO mice ([32]; and unpublished findings). Also, no further inhibition of aggregation was observed when platelets were treated with BAPTA and TRPC6 inhibitor when compared to BAPTA alone. This finding supports the specificity of the TRPC6 inhibitor; suggests that BAPTA chelated extracellular calcium including that which would have otherwise/presumably entered through TRPC6; and is consistent with the notion that TRPC6 does mediate calcium entry. We next sought to further define the role of TRPC6 in ROCE, as well as determine whether the aforementioned inhibitory effects on platelet function observed with the TRPC6 inhibitor are causally related to a blockade of ROCE. Indeed, it was found that the G_q_ agonist/TRPC6 activator [42] OAG stimulated ROCE, and that treatment with the TRPC6 inhibitor resulted in significant attenuation of ROCE. This is the first demonstration that TRPC6 regulates ROCE in human platelets, and indicate that there is a causal relationship between calcium homeostasis/entry and the inhibition of the various TPR-mediated platelet functional responses by the TRPC6 inhibitor. The fact that TRPC6 appears to play a selective role in TPR-mediated platelet function is rather a peculiar/interesting phenotype. This observation could be explained, in large, on the basis of the importance of G_q_-coupling to the individual receptors studied. Thus, given that TRPC6 is thought to be downstream of G_q_, it is expected that signaling pathways that are heavily dependent/reliant on G_q_ would be affected in presence of TRPC6 inhibitor. As for TPRs, work by Offermanns’ group have provided evidence that platelet shape change can be stimulated through the G_12/13_ pathway [18, 20], whereas aggregation and other responses are G_q_-mediated; which supports the notion that the latter is the primary pathway. On the other hand, there is substantial evidence that ADP and PAR signaling [43] are heavily dependent on G_i_ and G_13_, respectively. This is based on studies (using G_q_ knockout (KO) mice), in which it was concluded that PAR-mediated co-activation of G_12/13_ and G_i_-mediated signaling pathways is sufficient to induce platelet activation [26], and that G_13_ downstream of PAR has the capacity to increase intracellular calcium [44–46]. It is also possible that TRPC6/ROCE role in the various platelet functional responses may have an underlying temporal basis or be regulated via compartmentalization-dependent mechanisms; support of the latter hypothesis derives from previous studies that reported that functional compartmentalization of intracellular calcium with TRPC6 in particular does indeed exist [47, 48]. Having established the role of TRPC6 using “functional” assays, we next sought to confirm our findings biochemically by studying ERK and Akt phosphorylation. Indeed, our biochemical results revealed that TRPC6 regulates TPR-mediated activation of ERK and Akt, which is consistent with the platelet functional experiments, and the role of ERK and Akt in TPR signaling [49–51]. Of note, the “TPR-selective” phenotype observed supports the notion that the TRPC6 inhibitor is rather specific. To further investigate the mechanism of action of TRPC6 inhibitor, and exclude the possibility that it is acting as an antagonist for TPR, radioligand displacement binding studies were performed. Our results revealed that the TRPC6 inhibitor could not displace [^3^H]SQ29,548 from the platelet TPR binding sites, even when used at a concentration as high as five-fold of that that almost completely inhibited the aggregation response; which is consistent with it being an inhibitor for TRPC6 itself.

We next examined if TRPC6/ROCE regulates fibrin clot retraction, and found that TRPC6 inhibitor does indeed inhibit clot retraction. This data suggests that TRPC6 is involved in outside-in signaling. Finally, having established the capacity of TRPC6 to play a critical role in platelet function (*in vitro*) and in clot retraction, we next determined whether these effects would uphold under *in vivo* experimental models (e.g., antithrombotic activity). Hence, the mice were injected with TRPC6 inhibitor and subjected to the tail bleeding time assay. We found that inhibitor-treated mice exhibited impaired hemostasis as shown by their significantly prolonged bleeding times. The impaired hemostasis in these mice can be attributed to the defective TPR function, in the presence of the TRPC6 inhibitor. Given that it is well documented that calcium signaling plays an important role in the development of occlusive disorders, we sought to examine with its receptor-operated component via TRPC6 participates in thrombogenesis. Indeed, our data revealed that treatment with its inhibitor prolonged the time for occlusion, indicating that TRPC6 contributes to thrombus formation. This finding supports the notion that TRPC6 could be a target for novel anti-thrombotic agents, and is consistent with our previous findings [32] in which we showed that TRPC6 deletion in mice impairs hemostasis and thrombosis, as well as platelet aggregation in response activation by TRPs. To this end, a separate study showed that platelet functional responses were not affected by TRPC6 deletion in mice [52]. The differences between their findings and our human data with regards to platelet functional responses can be attributed to the different species and different experimental conditions. As for the differences in the *in vivo* phenotype, they are likely to be due to the experimental conditions, doses of agonist, genetic background, as well as the age of the animals used in these studies [53]. The latter issue is rather intriguing given that the role of TRPC6 has been indeed found to developmental stage dependent [53], something we were able to document in our laboratory (unpublished findings). Of note, our published mouse TRPC6 data [32] argues against species as an attributing factor since we observed that TRPC6 KO mice platelets exhibited a defect in their platelet functional responses.

Taken together, these data demonstrate that the mechanism by which TPR-mediates platelet aggregation, integrin activation, and secretion involves TRPC6 dependent CE. Furthermore, TRPC6 also regulates clot retraction, plays a physiologically relevant role in normal hemostasis, and in the pathogenesis of occlusive thrombi. We are the first to document the direct role of TRPC6 and ROCE in human platelets. Finally, our studies may define TRPC6 as a novel therapeutic target for managing multiple thrombosis-based disorders, albeit it may be associated with bleeding limitations, similar to all clinical antiplatelet agents.
